# Research Progress on the Functions and Mechanism of circRNA in Cisplatin Resistance in Tumors

**DOI:** 10.3389/fphar.2021.709324

**Published:** 2021-09-09

**Authors:** Qingchun Mu, Yue Lv, Chunmei Luo, Xiaojing Liu, Chunming Huang, Youcheng Xiu, Longguang Tang

**Affiliations:** ^1^ The People’s Hospital of Gaozhou, Gaozhou, China; ^2^ Department of Urology, The First Affiliated Hospital, Harbin Medical University, Harbin, China

**Keywords:** circular RNA, cisplatin resistance, cancer, non-coding RNA, gene

## Abstract

Cisplatin is a common chemotherapeutic drug that has been used to treat of numerous tumors, including testicular, lung, bladder, ovarian, liver and head and neck cancers. Although clinical chemotherapy based on cisplatin has shown a remarkable therapeutic effect, the resistance to cisplatin becomes increasingly obvious as a patient uses it for a prolonged period. It not only affects the prognosis of these tumors, but also causes the recurrence of cancer and decreases the overall survival rate. The development of cisplatin resistance involves several mechanisms, including DNA damage repair, ATP-binding cassette (ABC) transporter, autophagy, cancer stem cells (CSCs), epithelial–mesenchymal transition (EMT), and other related signaling pathways. Interestingly, these mechanisms have been found to be influenced by circular RNAs (circRNAs) to regulate tumor proliferation, invasion, chemosensitivity, and other biological behaviors in the tumor microenvironment (TME). In recent years, circRNAs in cisplatin resistance in tumors, especially lung cancer and gastric cancer, have gradually drawn peoples’ attention. This review summarizes recent studies on the functions and mechanisms of circRNAs in cisplatin resistance. We emphasize that circRNA can be used as a promising target gene to improve drug resistance and therapeutic efficacy.

## Introduction

### Cisplatin

**FIGURE 1:**
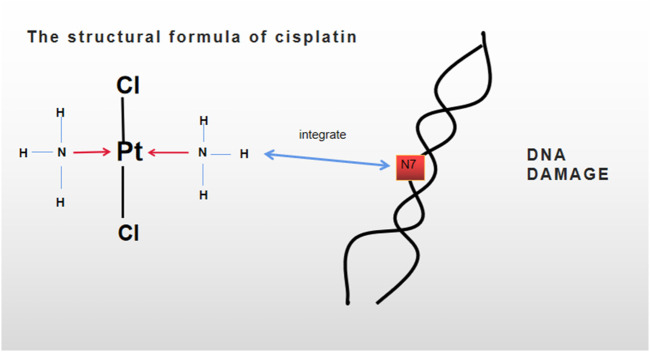
Molecular formula of cisplatin is Cl_2_H_4_N_2_Pt. DNA damage was caused by integrating cisplatin with the nucleophilic N7 locus of a purine base in a double-helix DNA.

Dr. Rosenborg accidentally discovered cisplatin in 1965 and predicted that it could inhibit cancer cell division (Rosenberg et al., 1965). About 50 years ago, cisplatin was approved by the US Food and Drug Administration (FDA) for the treatment of testicular cancer (Kartalou and Essigmann, 2001). Thereafter, cisplatin has become a common chemotherapeutic drug for numerous tumors, including testicular, lung, bladder, ovarian, liver, and head and neck cancers (Dasari and Tchounwou, 2014). It has numerous anticancer mechanisms. It is generally believed to interact with the nucleophilic N7 locus of purine base on DNA; as a result, DNA is damaged, and related signaling pathways are activated, thereby causing tumor cell apoptosis (Dasari and Tchounwou, 2014; Rocha et al., 2018; Ghosh, 2019) (Figure 1). With the continuous application of cisplatin in the chemotherapy of various tumors, its clinical efficacy has been found to be unsatisfactory. Cisplatin often develops resistance as patients use it for a prolonged period (Cocetta et al., 2019). Therefore, the cisplatin resistance mechanism of tumors has been widely studied to improve the survival rate and long-term prognosis of patients. For example, the accumulation of cisplatin and DNA compounds should be reduced by increasing the efflux or inhibiting the influx, lowering the toxicity of cisplatin with antioxidants (e.g., glutathione), enhancing DNA repair and DNA methylation, changing membrane protein transport, and activating or inactivating epithelial–mesenchymal transition (EMT) and other related pathways (Shen et al., 2012; Amable, 2016). Cisplatin resistance is also associated with cancer stem cells (CSCs). In addition, J.A.Ferreira et al. emphasized the functions of protein glycosylation in drug resistance (Ferreira et al., 2016). Besides, autophagy can promote the apoptosis of multidrug-resistant (MDR) cells, improve drug sensitivity, and protect cancer cells from the toxicity of chemotherapy drugs (Li et al., 2017). In recent years, increasing evidence has shown that the exocrine vesicles secreted from tumor cells can change the tumor microenvironment consequently, tumor growth is facilitated, and the resistance of chemotherapeutic drugs is promoted (Chiarugi and Cirri, 2016). Multiple mechanisms play roles in cisplatin resistance. These mechanisms are elaborated later.

### Circular RNAs (circRNAs)

**FIGURE 2:**
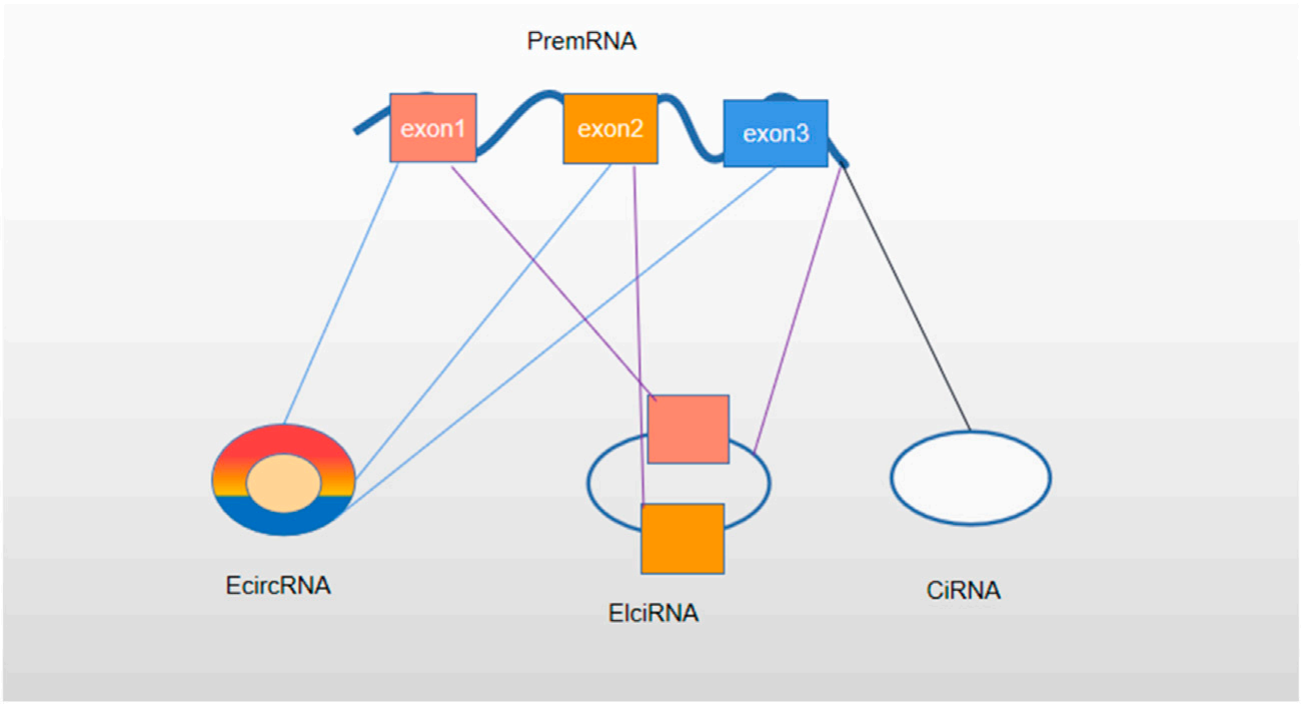
Classification of circRNA. Based on the composition, circRNAs are classified as exonic circRNAs (ecircRNAs), circular intron circRNAs (ciRNAs), and exon–intron circRNAs (EIciRNAs).

Noncoding RNAs (NcRNAs) can be divided into microRNAs (miRNAs), lncRNAs, and circRNAs (Iorio and Croce, 2017). CircRNAs were first discovered from a plant virus 40 years ago (Sanger et al., 1976). They have become a new hotspot in RNA research. CircRNAs are covalently closed-loop RNA molecules, which can splice the pre-RNA encoded by the coding gene through the interaction between reverse splicing and canonical splicing (Chen and Yang, 2015; Belousova et al., 2018). CircRNAs can be divided into three types, namely, exonic circRNAs, circular intron circRNAs, and exon–intron circRNAs (Meng et al., 2017) (Figure 2). They have no polyadenylation tail with 5′–3′ polarity (Chen and Yang, 2015). In mammals, circRNAs act as a sponge of miRNAs or combine with RNA-related proteins to form complexes to regulate gene expression and act as a gene regulator (Han et al., 2018). Some circRNAs can modulate gene transcription through RNA polymerase II in the gene promoter region. In addition, CircRNAs can also negatively regulate classical splicing through the interaction of the splicing factor muscleblind (MBL) (Su et al., 2019a). Recent studies have shown that circRNAs play an important role in the treatment of nervous system diseases, vascular diseases, and tumors (Qu et al., 2015). CircRNAs can be used as a biomarker of various cancers. They regulate tumor proliferation, invasion, chemosensitivity, and other biological behaviors by sponging miRNA or directly targeting a gene. Similar to lncRNAs, circRNAs and miRNAs also form a competing endogenous RNA (ceRNA) network (Su et al., 2019a; Verduci et al., 2019). The expression of circRNAs in tumor cells may be upregulated or downregulated compared with that in the surrounding normal tissues. Its effect may trigger tumor cells to respond positively or negatively to chemotherapeutic drugs.

Studies have gradually explored the regulatory effect of circRNAs on cisplatin resistance. This review summarizes recent studies on the functions and mechanisms of circRNAs in cisplatin resistance.

### Mechanisms

#### CeRNA net

**FIGURE 3:**
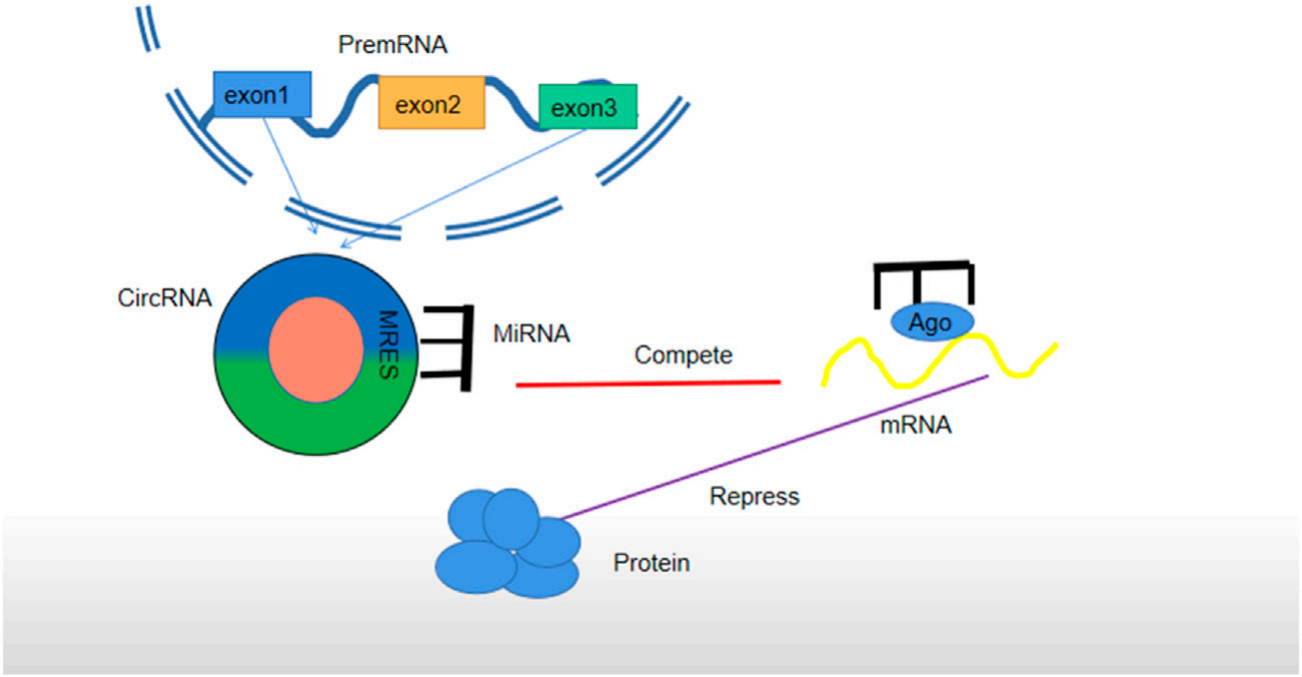
Regulatory mechanism of circRNA as ceRNA. CircRNA is cut off from a precursor RNA to sponge miRNA, block the expression of an miRNA-targeted gene (mRNA) in cells, and form a circRNA–miRNA–mRNA network. It has the same role in exosomes secreted by cells. Each miRNA can form a silent complex with an Argonaute (AGO) protein. An miRNA pairs with a target transcript, and the AGO protein promotes either target instability or transcriptional suppression.

CeRNA is an abbreviation of competing endogenous RNAs (Qi et al., 2015). CeRNAs comprise lncRNAs, circRNAs, protein-coding RNAs, tRNAs, rRNAs, and pseudogene RNAs (Qu et al., 2015). Theoretically, most ceRNAs have miRNA reaction elements (MREs). CeRNAs with the same MREs in single cells can regulate the transcription and translation of parental RNA by targeting the same or similar miRNA (An et al., 2017). The formation of a ceRNA network needs three basic conditions. First, a ceRNA should have a high expression level so that it is not affected by and interfered with the inhibition of downstream miRNAs on target genes. Second, only when the number of MREs is sufficient, circRNA can perform its biological function. The distribution and concentration of ceRNAs and miRNAs will also affect the ceRNA network. Third, different MREs have various effects on ceRNAs and miRNAs, which play a major role in ceRNA NET. Different circRNAs can bind to the same miRNA, but the nucleotide compositions of MREs may be partially different (Zhang et al., 2019) (Figure 3). In recent years, the studies on the mechanism of circRNA in cisplatin resistance are mostly based on the ceRNA network.

### Autophagy

**FIGURE 4:**
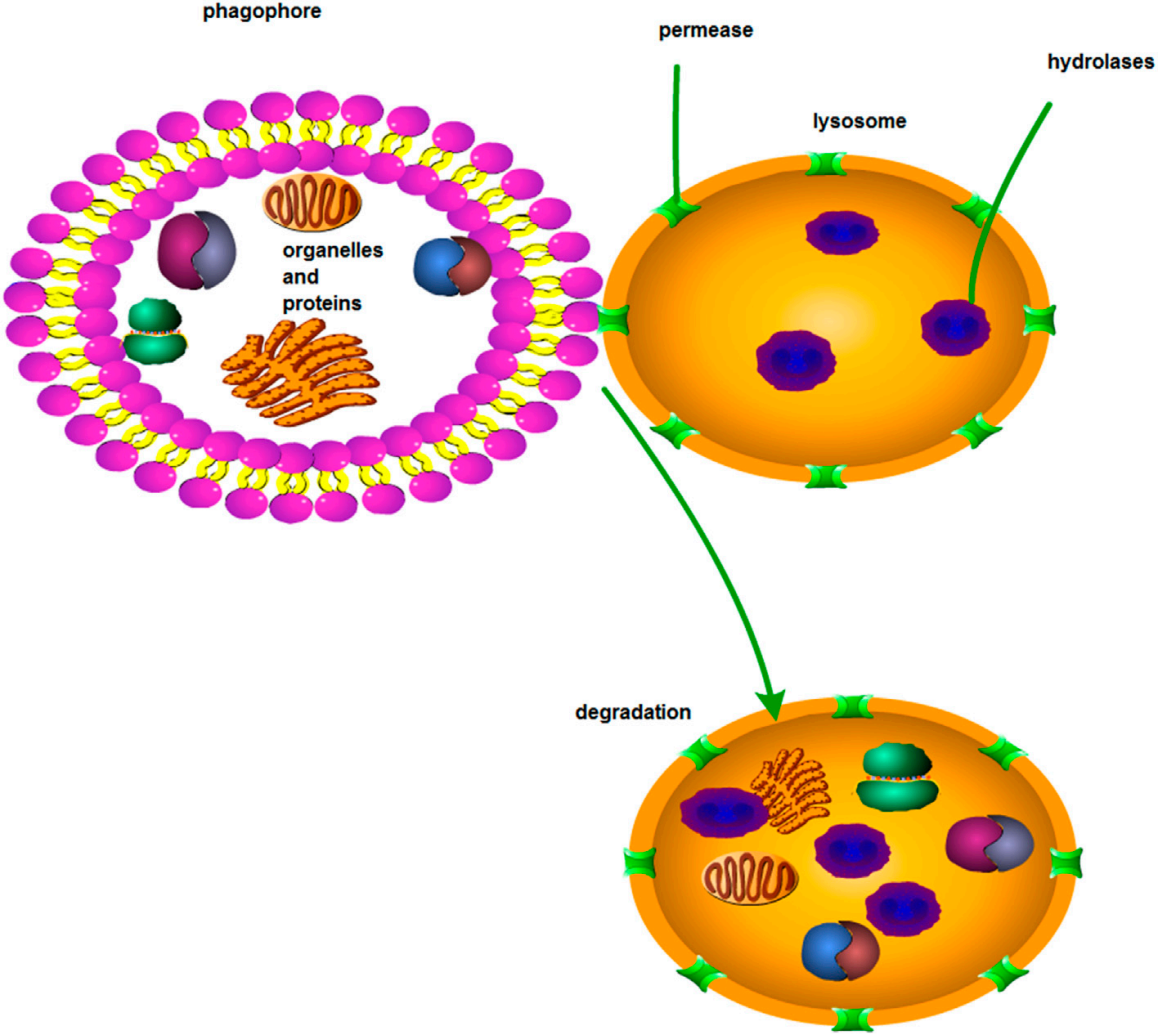
Macroautophagy. Through macroautophagy, substances in solute are transported to lysosomes via the autophagosome of a bilayer lipid.

Autophagy is a process through which starvation-induced lysosomes capture and degrade intracellular proteins and organelles and recycle intracellular components to maintain survival and metabolism (Amaravadi et al., 2016). Autophagy can be divided into macroautophagy, microautophagy, and concomitant factor-mediated autophagy (Guo et al., 2018). Macroautophagy is the most studied and classic form; in this process, autophagosomes transfer degraded substrates to lysosomes in the cytosol (Feng et al., 2014) (Figure 4). About 30 genes have been identified as autophagy-related genes (ATGs) in genetic analogues of many mammals and yeasts (Klionsky, 2012). For example, the deletion of Beclin-1, a mammalian gene of yeast ATG6, can promote tumorigenesis and support autophagy to inhibit tumor development (Qu et al., 2003). LC3 is an ubiquitin-like protein that combines with phosphatidylethanolamine (PE) to form a compound. This compound can interact with an ATG12-ATG5-ATG16L1 complex to confer the LC3–PE compound with the ability to produce an E3-like activity and participate in autophagy substrate degradation (Ichimiya et al., 2020). Autophagy disorders can lead to many diseases, such as Alzheimer’s disease, microbial infections, cardiomyopathy, diabetes, and even cancers (Levine and Kroemer, 2008; Sridhar et al., 2012).At early stages, autophagy is considered to inhibit cancer occurrence. However, at late stages, autophagy is the mechanism that promotes tumor cell survival. Autophagy plays a two-way regulation in tumor cells; thus, it has attracted considerable attention in tumor research (Sridhar et al., 2012). Many studies have also investigated the mechanism of autophagy in tumor chemoresistance. These studies have found that autophagy also plays a two-way role in the regulation of antitumor drugs. In the case of MDR, autophagy may be activated as a protective mechanism to protect tumor cells from the toxicity of chemotherapeutic drugs. However, autophagy can also induce tumor cells to die. Therefore, in this respect, autophagy can promote the sensitivity of chemotherapeutic drugs (Li et al., 2017). Most research results have shown that autophagy can promote cisplatin resistance in tumor cells. Studies have yet to determine whether autophagy can promote tumor cell apoptosis and the chemotherapeutic sensitivity of tumor cells remains unknown.

### ATP-Binding Cassette (ABC) Transporter

**FIGURE 5:**
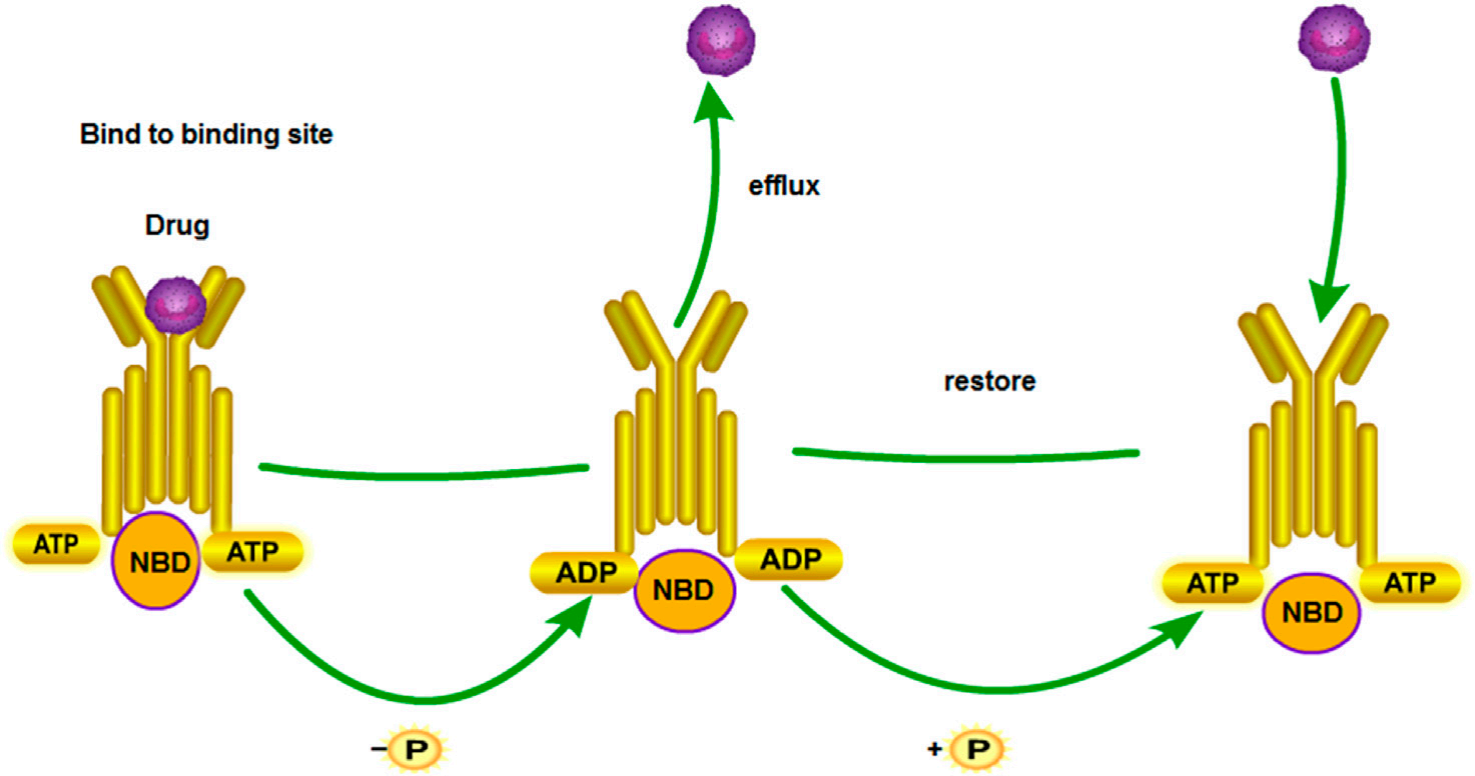
MDR1 drug transport mechanism. NBDs are nucleotide-binding domains. The substrate enters inside the protein. ATP binds to the binding site of NBD. ATP releases energy to change the conformation of transmembrane proteins and releases drugs out of the cell membrane. Subsequently, the next ATP is hydrolyzed to restore protein conformation. The above process is repeated.

ATP-binding cassette (ABC) transporters were discovered in the 1970s. They are energy-dependent transport systems of substrate-binding proteins (SBPs), which are activated by ATP hydrolysis (Theodoulou and Kerr, 2015). They can transport the solutes from the inside to the outside of cells (Figure 5). To date, 48 members of the ABC transporter protein family have been identified according to the sequence and structure of the ABC domain, and they are divided into seven A–G families. The ABC transporters can be mainly divided into three categories, namely, importers (prokaryotes), exporters (eukaryotes and prokaryotes), and ABCs repaired and translated by DNA. In short, importers import all kinds of molecules, whereas exporters export them (Paolini et al., 2015). Therefore, they influence the pharmacokinetics of chemotherapy (Paolini et al., 2015). Therefore, they influence the pharmacokinetics of chemotherapy. An ABC transporter identifies a series of antitumor drugs without structural correlation in cancer cells and transfers them outside the cell by using the energy of ATP hydrolysis (Stefan, 2019). Multidrug resistance protein 1 (MDR1) is also called P-glycoprotein. Its gene is known as ABCB1. The gene of breast cancer resistance protein (BCRP) is called ABCG2. The gene of multidrug resistance-related protein 1 (MRP1) is ABCC1. These three proteins are the most important ABC transporters in the mechanism of chemotherapeutic drug resistance in tumors (Hellsberg et al., 2015). CircRNA induces cisplatin resistance by regulating the expression of ABC transporter-related genes.

### Cancer Stem Cells (CSCs)

**FIGURE 6:**
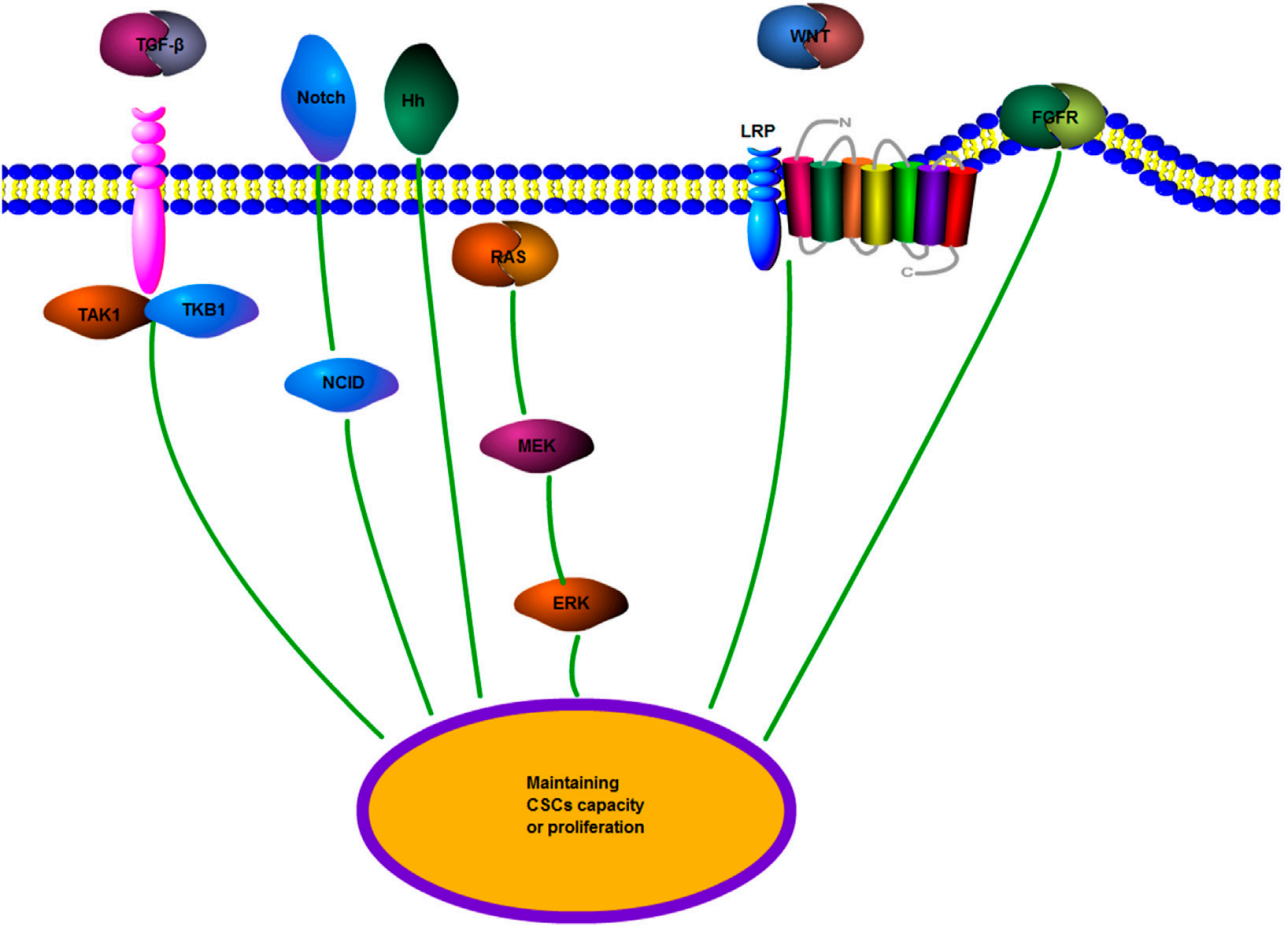
CSCs regulate drug resistance-related signaling pathways. TGF-β, transforming growth factor-β; Wnt/β-catenin; Hh, Hedgehog: Notch; FGFR, fibroblast growth factor receptor; and MEK, mitogen-activated protein kinase. Multiple signaling pathways promote the transformation of tumor cells to CSCs to maintain the features of CSCs or promote the growth and proliferation of CSCs.

Cancer stem cells (CSCs) are pluripotent tumor cells derived from normal stem cells, which have a self-renewal ability and a strong differentiation potential (Ajani et al., 2015; Lathia and Liu, 2017). The concept of CSCs has been widely recognized in the last decade, and CSCs are related to the growth and development of most malignant tumors (Chen et al., 2017). However, CSCs are generally insensitive to chemotherapeutic drugs. Although tumor cell chemoresistance occurs frequently, CSCs seem to be particularly resistant to chemotherapy. This resistance is due to the slower cell cycle of CSCs than that of many cancer cells targeted by chemotherapeutic drugs; their division speed is also not as fast as that of normal cells (Chang, 2016). CSCs are located in a special environment composed of fibroblasts and endothelial cells, mesenchymal cells, and immune cells. This environment is called a niche. Adjacent cells maintain CSCs through related signaling pathways and promote the endogenous drug resistance of CSCs (Prieto-Vila et al., 2017). Experiments have demonstrated that chemotherapy and radiotherapy can increase the quantity of CSCs and make tumor cells transform into CSCs (Chang, 2016). Moreover, several mechanisms, including EMT and ABC transporter, and signaling pathways including the Wnt pathway, participate in the drug resistance of CSCs. They are introduced in the following sections (Prieto-Vila et al., 2017). Therefore, to explore the role of CSCs in cisplatin resistance in tumors, pathways including fibroblast growth factor receptor (FGFR) or transforming growth factor-β (TGF-β) should be investigated to explore the role of CSCs in cisplatin resistance in tumors (Robey et al., 2018) (Figure 6). In discussing the role of circRNAs in cisplatin resistance, studies on the interaction of multiple mechanisms, including CSCs, are presented to discuss the role of circRNAs in cisplatin resistance.

### Exosomes

Exosomes are nanosized bilayer vesicles composed of extracellular lipids, which contain some biomolecules, such as lipids, proteins, and nucleic acids. Exosomes were first believed to exclude useless cellular components; later, they were found to be involved in many physiological and pathological processes, even cancers (He et al., 2018). In addition, primary tumor biomarkers are transferred by exosomes to distant organs to achieve tumor metastasis (García-Olmo et al., 1999). These molecular markers include miRNAs, lncRNAs, and circRNAs. Thus, the effect of exosomes on tumors should be further studied. The drug resistance of exosomes in tumors has been extensively studied. Exosomes can promote tumor drug resistance through the following ways: firstly, exocrine molecular substances can compete with some anticancer chemotherapeutic drugs and bind to oncogenic targets to produce drug resistance; Secondly, exosomes transfer the drug resistance of drug-resistant cells to sensitive cells; Thirdly, tumor cells secrete chemotherapeutic drugs outside cells through exosomes (Zhang et al., 2018a). This principle applies to cisplatin resistance in tumor cells. CircRNAs secreted by exosomes promote related tumor cells to produce resistance to chemotherapeutic drugs. Similarly, circRNAs secreted by exosomes can regulate other targeted genes or other pathways, such as CSCs and Wnt, to regulate the biological characteristics of tumor cells.

### DNA Damage Repair

Chemotherapeutic drugs, such as cisplatin, can damage the DNA. Accordingly, a mechanism called DNA damage repair (DDR) maintains the stability of DNA (including DNA in cancer cells) in our body. These damages are repaired through many methods, including nucleotide excision repair (NER), mismatch repair, base excision repair, and homology-directed repair (Gavande et al., 2016). The key genes encoding DNA damage response and DNA repair include BRCA1 and BRCA2 germline mutation, which leads to cancer susceptibility syndrome. The exposure of these tissues to carcinogens makes them more likely to become cancerous (Brown et al., 2017). In terms of drug resistance, excision repair cross-complementing (ERCC1) forms an ERCC1–XPF enzyme complex, which can repair the DNA damage caused by chemotherapeutic drugs via NER. ERCC1 overexpression is negatively correlated with the clinical outcome of platinum therapy (Zheng, 2017). Therefore, ERCC1 can be used as an indicator of cisplatin resistance, indicating that it plays a role in DNA repair (Huang et al., 2019a). DNA polymerase ζ plays a role in the microhomology-mediated repair of break-induced replication. REV3L is the largest catalytic subunit in DNA polymerase ζ, indicating that it also participates in DNA repair (Martin and Wood, 2019). Therefore, REV3L overexpression regulated by circRNAs can promote cisplatin resistance (Pang et al., 2020).

### Epithelial–Mesenchymal Transition

**FIGURE 7:**
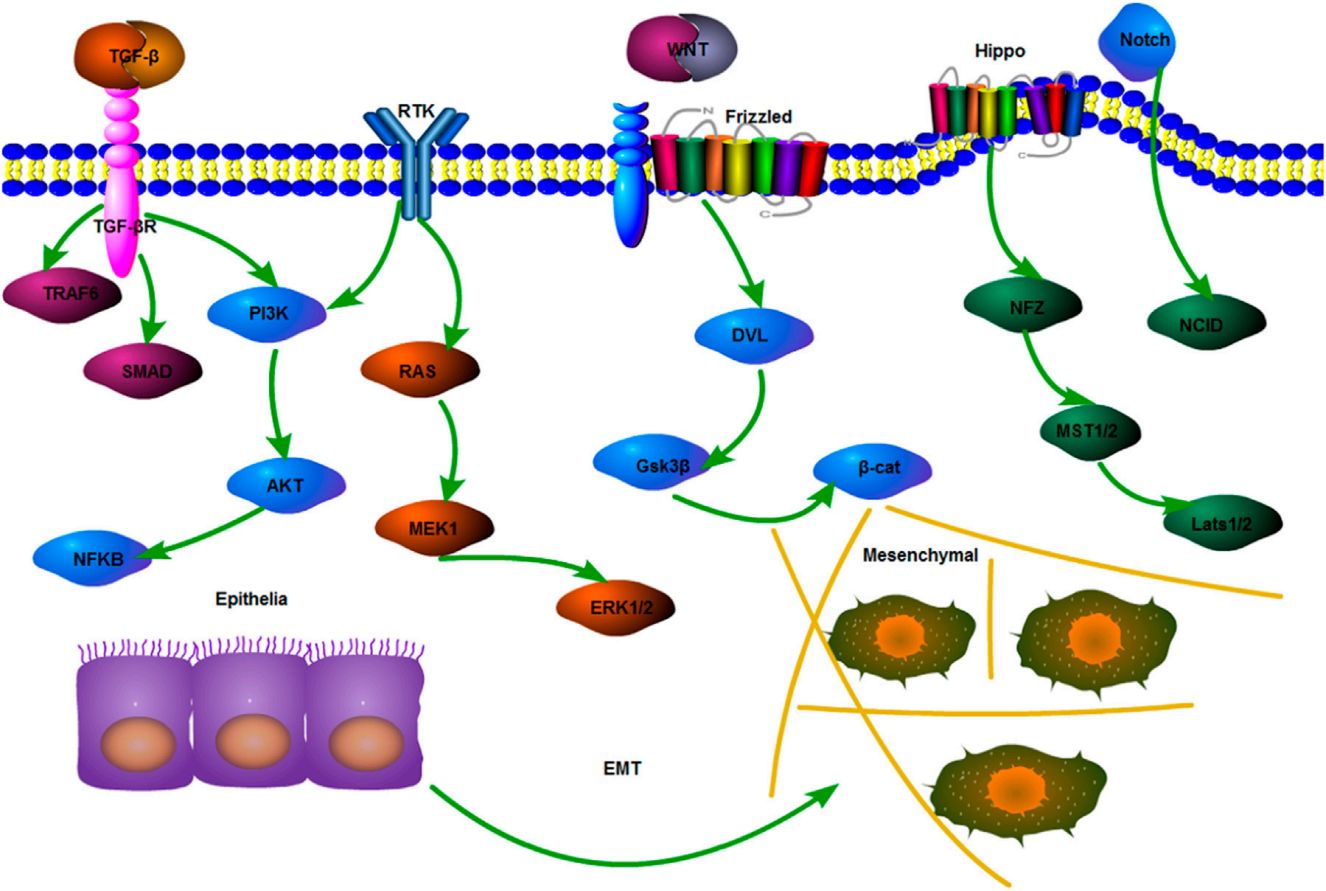
Mechanisms of EMT-related pathways. Mechanisms of EMT-related pathways include the most classical pathway, that is, TGF-β regulates the expression of a downstream cell attachment gene to lower by activating the Smad complex. Other pathways that can maintain and promote EMT include PI3K/Akt, Wnt, Hippo, and Notch.

The lineage transition between epithelial and mesenchymal cells shows that polarized epithelial cells lose their adhesion properties and obtain a mesenchymal phenotype; this process is called EMT (Chen et al., 2017). The role of EMT in tumor drug resistance has been gradually recognized and explored. In the mouse model of Fischer et al., cyclophosphamide treatment significantly reduces the amount of primary epithelial tumors, but the amount of tumor cells with a positive EMT is not significantly reduced. This finding indicates that EMT may be involved in the formation of drug resistance, including TGF and other factors in an EMT-mediated signaling pathway (Du and Shim, 2016). In colon cancer cells, TGF-β upregulation can promote EMT and Adriamycin resistance (Li et al., 2015). As mentioned before, CSCs can interact with EMT to regulate the biological characteristics of tumors. They are related to multiple pathways. Similarly, EMT also includes TGF‐β, Notch, Wnt, KRAS, and phosphatidylinositol 3-kinase (PI3K) pathways (Singh et al., 2018) (Figure 7).

### Signaling Pathways

#### Wnt Signaling Pathway

The Wnt signaling pathway is involved in the embryonic development and homeostasis of normal adults and many normal processes. The human Wnt protein family contains 19 glycoproteins. This signaling pathway is not only involved in the proliferation, renewal, and survival of normal human cells but also implicated in diabetes, Parkinson’s disease, and cancers (Duchartre et al., 2016). The Wnt signaling pathway can be roughly divided into two categories, namely, β-catenin-dependent classical type and nonclassical type. The β-catenin-dependent classical pathway is the most frequently studied pathway. APC mutations in CRC have been discovered for many years. A study on human CRC specimens and mouse models has found that different types of APC mutations can activate different Wnt/β-catenin classical pathways to promote tumor formation and proliferation (Zhan et al., 2017). The Wnt signaling pathway is necessary for most stem cells, including normal tissue stem cells or tumor stem cells (Nusse and Clevers, 2017). It also plays a role in tumor invasion and metastasis and related chemoresistance.

#### KRAS

The KRAS pathway is involved in the formation of cisplatin resistance. KRAS is the most common mutation of the RAS gene, which plays a key role in the occurrence of malignant tumors (Chen et al., 2019a). KRAS is a signaling pathway that regulates cell proliferation, apoptosis, and other biological activities through linear sensing signal molecules from the cell membrane to the nucleus (Liu et al., 2019). It also participates in cell cycle. When working against cancers, it can promote tumor cells to go into the S phase from the G1 phase and prevent cell apoptosis induced by remaining at the G0 phase (Nussinov et al., 2017). Therefore, KRAS activation can prevent cell apoptosis induced by cisplatin and other chemotherapeutic drugs.

#### Phosphatidylinositol 3-Kinase

PI3K regulates the signal transduction of human insulin and the proliferation and survival of cells. In addition, the mutated PI3K pathway controls the growth and proliferation of cancer cells (Lien et al., 2017). Epidermal growth factor receptor (EGFR) is a receptor tyrosine kinase that can phosphorylate downstream signaling molecules. EGFR activation can trigger multiple downstream signal transduction pathways. The PI3K/Akt/mTOR pathway is one of the classical pathways. It regulates cell division, migration, proliferation, and differentiation (Li et al., 2016). CC-chemokine ligand 2 (CCL2) secreted from tumor-associated macrophages promotes tamoxifen resistance by activating the PI3K pathway (Li et al., 2020a). Therefore, the mechanism of the PI3K pathway in chemotherapy resistance in tumors has attracted increasing attention.

**FIGURE 8:**
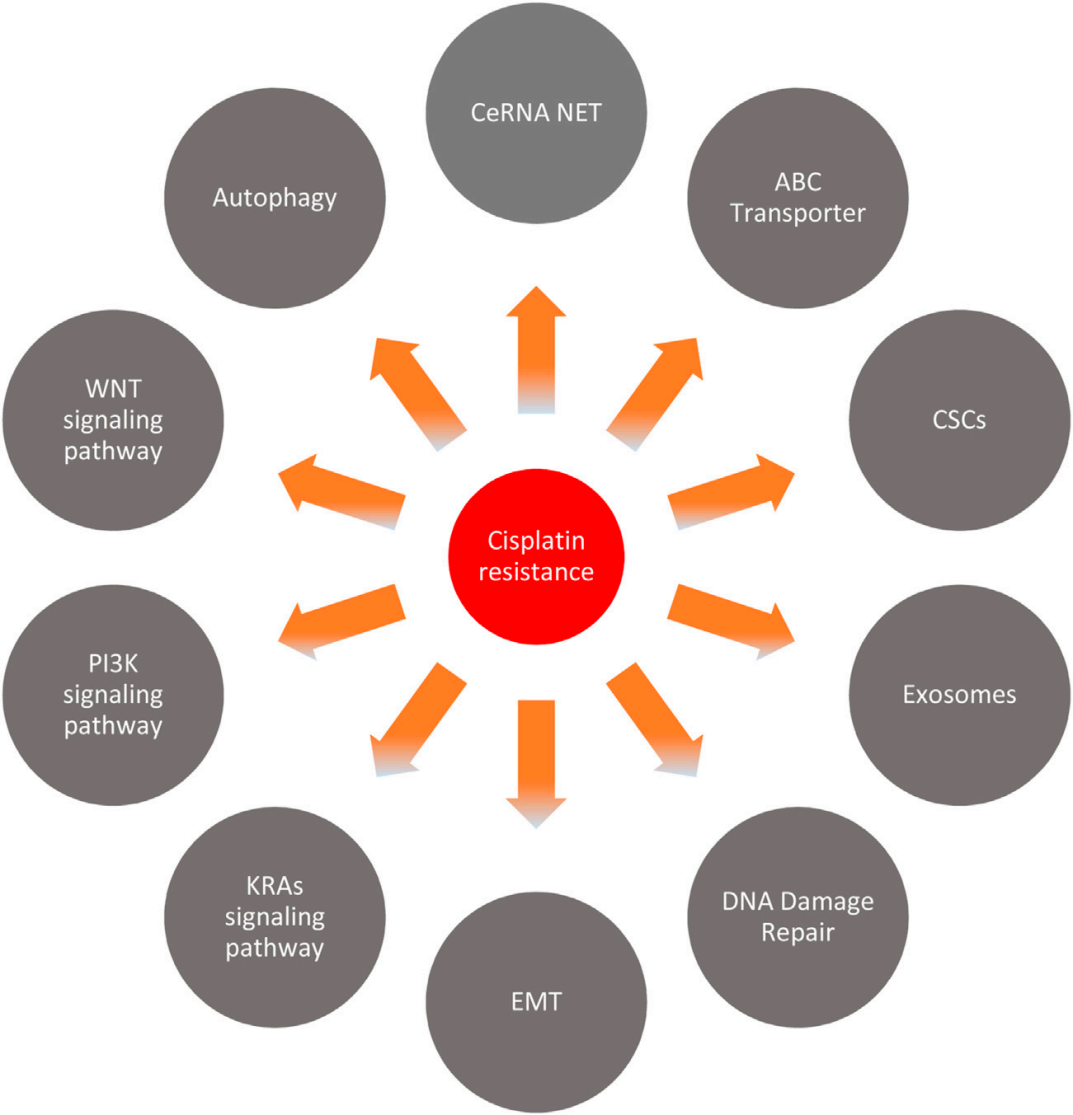
Cisplatin resistance mechanism.

Many mechanisms regulate the cisplatin resistance of circRNA in several cancers (Figure 8). We discuss these mechanisms in several kinds of cancer in the following sections.

### Cisplatin Resistance in Cancer

#### Lung Cancer

**FIGURE 9:**
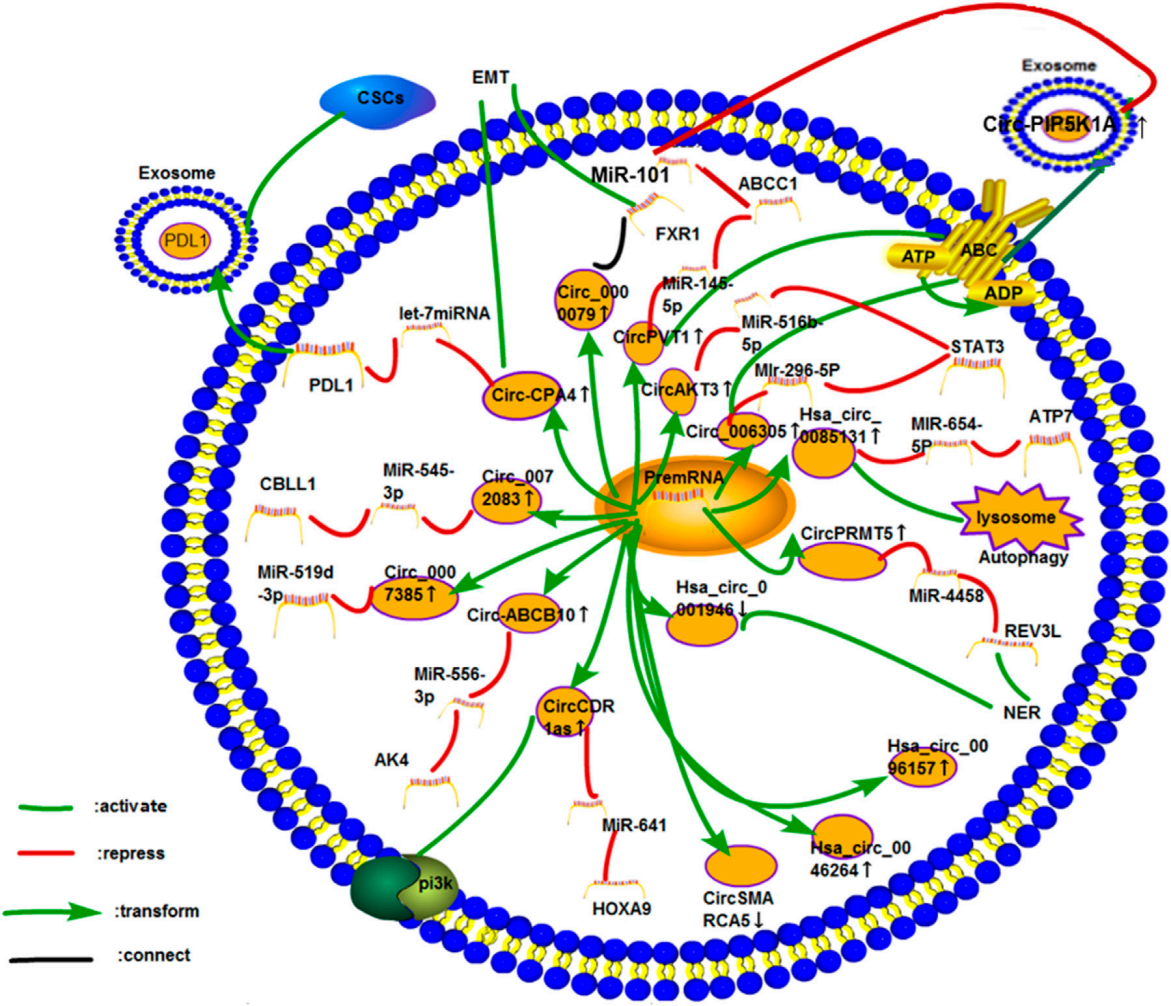
Cisplatin resistance mechanism of circRNA in lung cancer. So far, 16 circRNAs have been studied in lung cancer. The mechanisms of cisplatin resistance in these articles include the most common ceRNA NET, exosomes, PI3K pathway, autophagy, ABC transporter, DDR (NER), and CSCs. Three circRNAs, namely, hsa_circ_0096157, hsa_circ_0046264, and CircAMARCA5, play roles in cisplatin resistance in lung cancer. However, their specific mechanisms have not been explored.

Lung cancer is the leading cause of death in men and the second leading cause of death in women worldwide (Torre et al., 2016). Many studies have investigated the efficacy of chemotherapy after lung cancer surgery, and some of them have proven the benefits of cisplatin-based neoadjuvant chemotherapy (Nagasaka and Gadgeel, 2018). (Figure 9).

CeRNA NET has been extensively studied in the drug resistance of lung cancer cells. Circ_0072083 could enhance the resistance of non-small cell lung cancer (NSCLC) to cisplatin via the miR-545-3p/CBLL1 axis. MiR-545-3p could significantly inhibit the proliferation, cell cycle, and invasion of NSCLC cells and promote cisplatin-induced apoptosis (Li et al., 2020b). The expression of circ_0007385 in NSCLC tissues and cell lines was higher than that in adjacent normal tissues, and this finding was associated with low overall survival (OS) rate. Circ_0007385 could regulate the high expression of HMGB1 in lung cancer tissues by sponging miR-519d-3p, so as to promote the proliferation, invasion, and cisplatin resistance of tumors (Ye et al., 2020).

As early as 2017, circ-ABCB10 has been reported to promote the proliferation and invasion of breast cancer cells by sponging miR-1271 (Liang et al., 2017). The knockdown of circ-ABCB10 expression could upregulate the sensitivity of miR-556-3p to cisplatin in lung cancer cells, whereas upregulating AK4 could reverse this phenomenon (Wu et al., 2020). CircAKT3 was revealed to inhibit the homeostasis of glycolysis through miR-516-5P/STAT3 to increase the cisplatin resistance of lung cancer cells (Xu et al., 2020a). Yu Dong et al. had reported that the expression of circ_0076305 was upregulated in drug-resistant NSCLC. Circ_0076305 could enhance the STAT3 expression by sponging miR-296-5P and the resistance of NSCLC to cisplatin (Dong et al., 2019). Western blot analysis showed that upregulating circ_0076305 could improve the protein expression of P-GP (MDR1) and MRP1. As mentioned before, MDR1 and MRP1 are ABC transporters. The ABC transporter has also been reported to be involved in lung cancer. CircPVT1, a ceRNA of miR-145-5p, could regulate the expression of ABCC1 and lower the sensitivity of pulmonary adenocarcinoma to pemetrexed and cisplatin (Zheng and Xu, 2020). Huasong Lu et al. detected the specimens of patients with NSCLC through PCR and found that the expression of circPVT1in the drug-resistant group was higher than that in the drug-sensitivity group when patients were treated with a combination of cisplatin and gemcitabine. The high expression of circPVT1 was also associated with the survival rate of patients (Lu et al., 2020a).

Few studies have focused on exosomes in lung cancer. Circ-CPA4 may regulate PD-L1 by activating the miRNA of let-7 to promote the proliferation, invasion, EMT, and cisplatin resistance of lung cancer cells. Circ-CPA4 knockdown could lower the expression of cyclin D1 and Bcl-2 and reduce the tumorigenesis in mice during heterotransplantation. Some studies had shown that exosomes of NSCLC containing PD-L1 could form immune escape by increasing the mRNA levels of stem cell-related signal and inactivating CD8 T cells in order to enhance cisplatin resistance (Hong et al., 2020). Anti-PD-1/PD-L1 has been widely used to treat solid tumors, but its curative effect is unsatisfactory (Lei et al., 2020). Therefore, PD-1/PD-L1 blocking reagent can be used in combination with cisplatin and other chemotherapeutic drugs. Na Shao et al. demonstrated that exosomal circ_PIP5K1A could promote cisplatin resistance by targeting miR-101 and consequently modulating ABCC1 expression in NSCLC (Shao et al., 2021).

Some studies have investigated the role of EMT-related genes in tumor drug resistance. Chen et al. revealed that the level of circ_0000079 (CiR79) in patients with NSCLC, especially those who were resistant to cisplatin, was significantly decreased. Low circ_0000079 levels were correlated with a low OS rate. Circ_0000079 could block the formation of the FXR1/PRCKI complex by combining with FXR1 to inhibit cell invasion and improve the chemosensitivity of NSCLC. FXR1/PRKCI-mediated glycogen synthesizes kinase 3β and activator protein-1 phosphorylation to inhibit a snail protein level (Chen et al., 2020a). Except NSCLC, FXR1 is related to poor prognosis in some cancers, including ovarian cancer, breast cancer, and head and neck squamous carcinoma. It is related to the PKC expression and the iota and epithelial transition (Raheja and Gandhi, 2016). The snail gene plays an important role in EMT in tumors and the proliferation of tumor cells (Chen et al., 2020a).

In the previous description of circ-CPA4, stem cells are mentioned. The role of CSCs in the cisplatin resistance of lung cancer cannot be ignored. The circRNA CDR1as/miR-641/HOXA9 axis could regulate the apoptosis of stem cells and enhance cisplatin resistance in NSCLC. In cisplatin-resistant NSCLC cells, the overexpression of circRNA CDR1as had been reported to increase the mRNA levels of stem cell signals (SOX2, OCT4, and Nanog). This phenomenon could be reversed by upregulating miR-641 and downregulating HOXA9 (Zhao et al., 2020).

Autophagy helps tumor cells respond to intracellular and environmental stresses, such as hypoxia, malnutrition, and cancer chemotherapy. Autophagy inhibition can improve the therapeutic effect of patients with advanced cancers (Amaravadi et al., 2019). The role of the ceRNA network in regulating tumor autophagy in chemoresistance has been confirmed. Hsa_circ_0085131 could be used as a ceRNA of miR-654-5P to release ATP7, which could enhance the autophagy and cisplatin resistance of lung cancer cells (Kong, 2020).

DDR can be completed by NER. Hsa_circ_0001946 in NSCLC cells was downregulated, and it could inhibit cell proliferation, invasion, and migration. The downregulation of hsa_circ_0001946 could activate the NER signaling pathway and reduce the sensitivity of cisplatin (Huang et al., 2019b). In addition, the expression of circRNA PRMT5 in cisplatin-resistant NSCLC had been revealed to lower its sensitivity to cisplatin. Moreover, the inhibition on the sensitivity of NSCLC to cisplatin was recovered through miR-4458 inhibition or REV3L upregulation by silencing circRNA PRMT5 (Pang et al., 2020). Therefore, studying the REV3L upstream genes in cisplatin resistance is of great significance.

Yuqiang Mao et al. found that circRNA CDR1as could enhance the pemetrexed and cisplatin resistance of lung adenocarcinoma in neoadjuvant chemotherapy through the EGFR/PI3K signaling pathway. Western blot analysis shows that the expression of EGFR and PI3K proteins in drug-resistant cell lines increases. In addition, the silencing of circRNA CDR1as could lead to the sensitivity of lung cancer cells to chemotherapeutic drugs. This effect can be blocked by upregulating EGFR (Mao and Xu, 2020). Huasong Lu et al. also reported that hsa_circ_0096157 could regulate the cisplatin resistance of NSCLC by regulating cell proliferation, apoptosis, and cell cycle (Lu et al., 2020b). The expression of circ-SMARC5 was found to reduce the proliferation of NSCLC and improve its chemosensitivity to cisplatin and gemcitabine (Tong, 2020). Liu et al. found that the expression of hsa_circ_0046264 in lung cancer was significantly upregulated, and this upregulation was associated with tumor proliferation, invasion, and stage. The expression of hsa-circ_0046264 was high in cisplatin-resistant cell lines (Liu et al., 2020a). The cisplatin resistance mechanism of these circRNAs has not been discussed and should be further studied.

### Gastric Cancer

**FIGURE 10:**
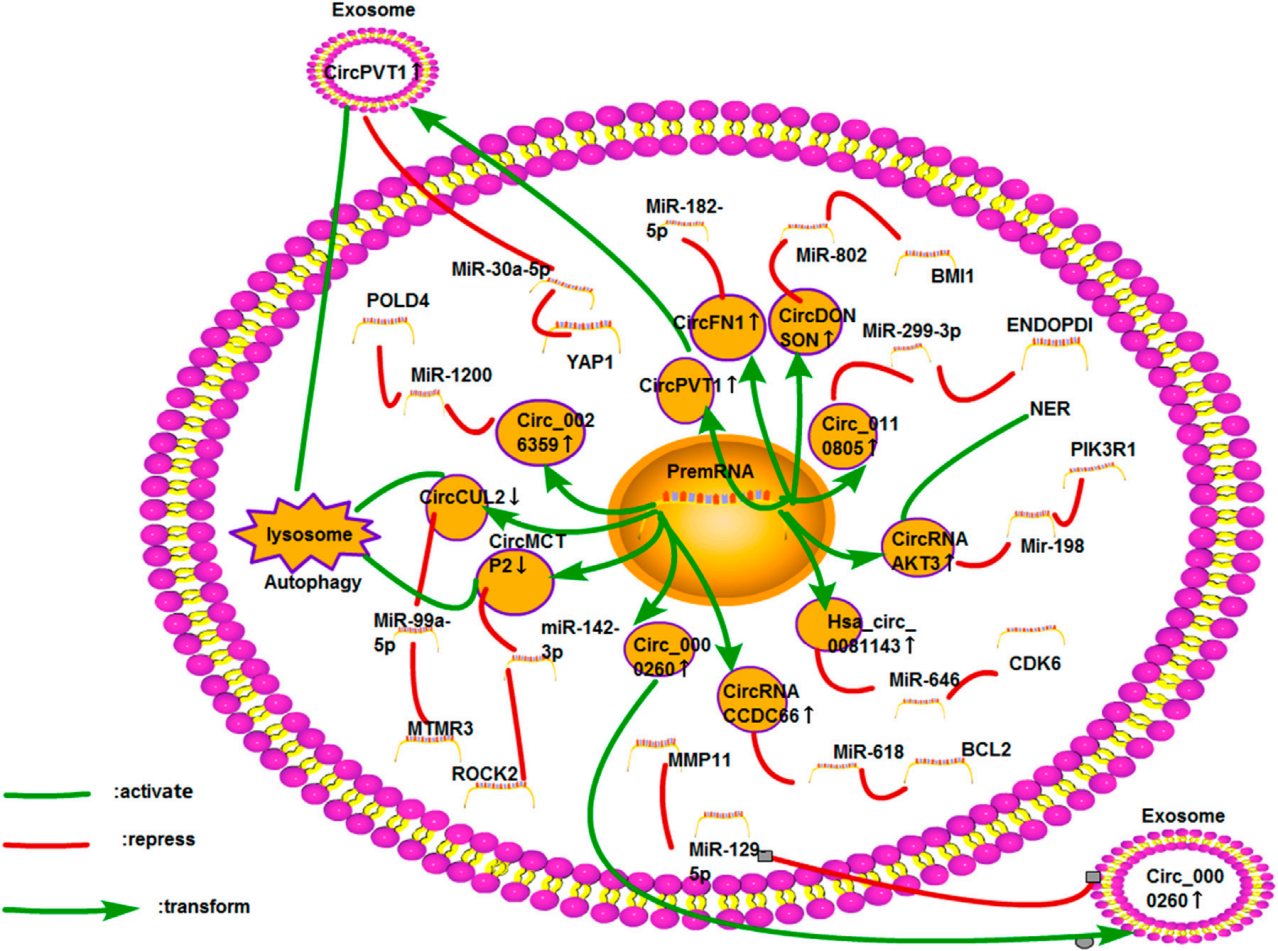
Cisplatin resistance mechanism of circRNA in GC. At present, 11 circRNAs are found to be related to cisplatin resistance in GC. The mechanisms involved include ceRNA NET, exosomes, autophagy, and DNA damage and repair.

GC is the fifth-most common cancer in the world. Generally, most patients are diagnosed with GC at an advanced stage. Systemic chemotherapy is still the main treatment for patients with advanced GC (Wagner et al., 2017). Cisplatin is commonly used in clinical chemotherapy. Some chemotherapy regimens, including docetaxel, cisplatin, and 5-fluorouracil, have been proven to be effective in the treatment of GC (Li et al., 2019a). (Figure 10).

Many studies have investigated the effect of circRNAs on the cisplatin resistance of GC, and most of them are based on the ceRNA network. Minghui Xue et al. reported that the expression of hsa_circ_0081143 in GC cells was upregulated, which was related to the proliferation, invasion, and metastasis of tumors. Hsa_circ_0081143 can also enhance the cisplatin resistance of GC cells via the miR-646/CDK6 pathway (Xue et al., 2019a). Circ_0110805 was overexpressed in cisplatin-resistant GC tissues or cells. Cisplatin resistance could be enhanced through the miR-299-3P/*ENDOPDI* axis (Yang et al., 2020a). The ceRNA network for circRNA /miR-646/ miR-299-3P should be further studied.

Lower OS rate, recurrence-free survival, and cisplatin resistance rate were associated with high circ_0026359 levels. Circ_0026359 could enhance the cisplatin resistance of GC via the miR-1200/POLD4 pathway (Zhang et al., 2020a). CircRNA 0001785 was shown to promote the proliferation of osteosarcoma and inhibit its apoptosis through the miR-1200/HOXB2 axis (Li et al., 2019b). Therefore, we should explore whether circRNA 0001785 also has a chemotherapy mechanism, including cisplatin resistance, in osteosarcoma or GC. Xiao Xu Huang et al. first found that circFN1 could improve the survival rate and cisplatin resistance of GC cells by sponging miR-182-5p; thus, it could be used as a new therapeutic target for cisplatin-resistant patients with GC (Huang et al., 2020). Cheng Yang et al. had found that circFN1 could promote the resistance of liver cancer cells to sorafenib (Yang et al., 2020b). Quantitative reverse transcription PCR (qRT-PCR) showed that the expression level of circRNA CCD66 in GC tissues was higher than that in normal tissues, especially in cisplatin-resistant cell lines. In terms of the mechanism, circRNA CCD66 could inhibit apoptosis by targeting the miR-618/BCL2 axis (Zhang et al., 2020b). Recently, circRNA CCD66 had been found to promote the resistance of CRC to oxaliplatin by regulating autophagy (Lin et al., 2020). CircDONSON was upregulated in GC cells. The transfection of si-circDONSON could lower the IC_50_ value of DDP, indicating that circDONSON could promote cisplatin resistance of GC. Studies had shown that circDONSON inhibited apoptosis and cisplatin resistance by sponging miR-802 (Liu et al., 2020b).

Studies have also investigated autophagy in GC. QRT-PCR showed that the level of circCUL2 in GC tissues significantly lowered than that in normal tissues. In GC cells overexpressing circCUL2, the levels of autophagosomes were low. Western blot analysis showed that BCL-2 expression is similar to the trend of autophagosomes. CircCUL2 was revealed to regulate the sensitivity of cisplatin via the miR-142-3P/ROCK2 axis (Peng et al., 2020). In comparison with chemosensitive cell lines, downregulateing the expression of circMCTP2 could activate autophagy in cisplatin-resistant GC cells. The heterotransplantation model showed the same trend. The overexpression of circMCTP2 could inhibit autophagy and enhance cisplatin sensitivity via the miR-99a-5p/MTMR3 axis (Sun et al., 2020a). Therefore, autophagy can enhance cisplatin resistance. In terms of the exosomes of GC cells, autophagy is also involved. In GC, circPVT1 from exosomes could regulate autophagy through the miR-30A-5P/YAP1 axis and enhance cisplatin resistance (Yao, 2020). The abnormal expression of YAP1 in HCC, GC, CRC, and lung cancer is regarded as a sign of poor prognosis (Shibata et al., 2018). Therefore, the regulation of the drug resistance of tumor cells through the ceRNA network should be studied by targeting the YAP1 gene. In addition, the expression of circ_0000260 in cisplatin-resistant gastric adenocarcinoma tissues and exosomes from the serum was higher than that in sensitive tumor tissues. A dual-luciferase experiment had demonstrated that miR-129-5p was the downstream target gene of circ_0000260, and MMP11 was the direct target gene of miR-129-5p (Liu et al., 2020c).

The expression of circAKT3 was upregulated in cisplatin-resistant GC, which had a strong invasive capacity. Through detecting the levels of caspase-3 and BRCA1, circAKT3 could inhibit apoptosis and promote DDR. On the basis of miRanda, RNAhybrid, GeneChip, and other databases, Huang Xiaoxu et al. revealed that circAKT3 could regulate the expression of PI3KR1 by sponging miR-198 (Huang et al., 2019a). As mentioned above, circAKT3 may enhance the cisplatin resistance of lung cancer cells (Xu et al., 2020a). This phenomenon indicates that circAKT3 may be a valuable gene for studying other cancers. However, the expression of circAKT3 was downregulated in renal clear cell cancer, thereby reducing its metastatic ability through the miR-296-3P/E-cadherin signaling pathway (Xue et al., 2019b). However, the study does not explore the next study on its antitumor effect.

### Bladder Cancer

**FIGURE 11:**
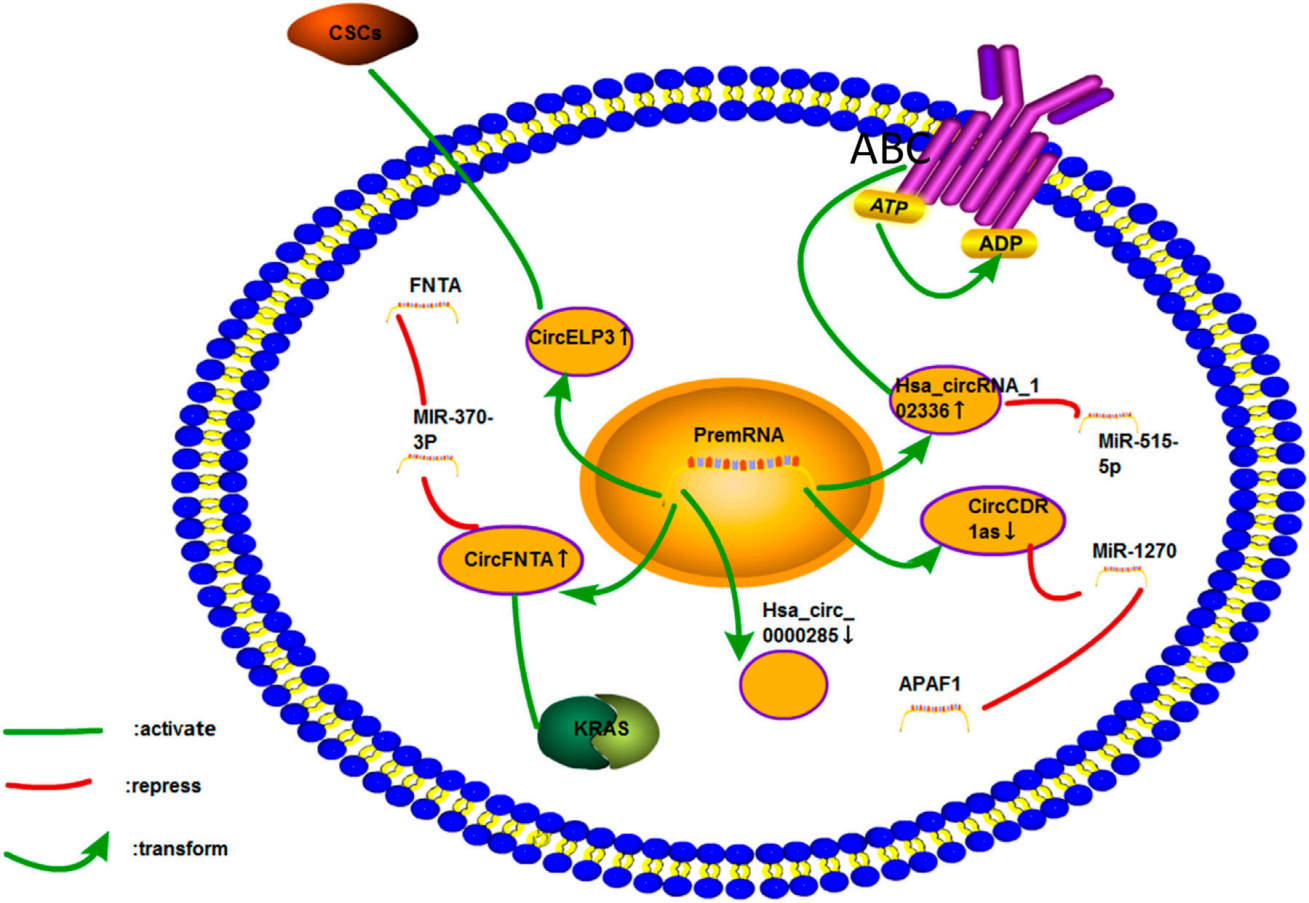
Cisplatin resistance mechanism of circRNA in bladder cancer. Only five circRNAs associated with the cisplatin resistance of bladder cancer have been studied. The mechanisms involved include ceRNA NET, CSCs, ABC transporter, and KRAS pathways.

Bladder cancer is the ninth-most common cancer in the world (Gong et al., 2020a). Cisplatin-based chemotherapy has been used to treat muscle-invasive bladder cancer. However, its curative effect remains unsatisfactory (Schardt et al., 2019). (Figure 11).

The circRNA CDR1as could promote the resistance of lung cancer to chemotherapeutic drugs (Mao and Xu, 2020; Zhao et al., 2020). It could also promote the apoptosis and sensitivity of bladder cancer cells to cisplatin by sponging miR-1270 and regulating APAF1 (Yuan et al., 2019). Thus, this circRNA seems to have different roles in different cancers. Therefore, its role in the pathogenesis and chemoresistance of different types of cancers should be investigated. The ABC transporter plays an essential role in the cisplatin resistance of bladder cancer. On the basis of the GSE92675 database, Pengfeng Gong et al. found that the upregulation of circ_102336 expression in bladder cancer tissues and cells was associated with tumor proliferation and low survival rate. Circ_102336 could also promote the cisplatin resistance of bladder cancer cells and regulate apoptosis and ABC transport route by sponging miR-515-5p (Gong et al., 2020a). CircRNAs could also promote oxaliplatin resistance in GC by targeting miR-515-5p (Zhong et al., 2020). The androgen acceptor (AR) represses ADAR2 to enhance the expression of circFNTA. CircFNTA could enhance cisplatin resistance of bladder cancer cells through the miR-370-3P/FNTA pathway and activating the KRAS pathway (Chen et al., 2020b).

Yinjie Su et al. found that hypoxia could increase the level of circELP3. They also found that the high expression of circELP3 could inhibit the apoptosis of cancer cells and promote cisplatin resistance by targeting tumor stem-like cells (Su et al., 2019b). Hypoxia could promote the expression of some ncRNAs, so as to enhance the proliferation of bladder cancer cells. Under hypoxic condition, the expression of hsa_circRNA_403658 in bladder cancer was increased, and hsa_circRNA_403658 activated IDHA-mediated aerobic glycolysis to promote the proliferation of bladder cancer cells (Wei et al., 2019). Hypoxia could also induce circNRIP1 to increase its expression and improve the resistance of GC to 5-fluorouracil (Xu et al., 2020b). A previous study involving qPCR showed that hsa_circ_0000285 could improve the sensitivity of patients with bladder cancer to cisplatin, which may be related to the stage, differentiation, and metastasis of tumors (Chi et al., 2019). However, the study did not explore the specific mechanism of drug resistance. Further studies on the mechanisms involved in some articles should be performed.

### Liver Cancer

**FIGURE 12:**
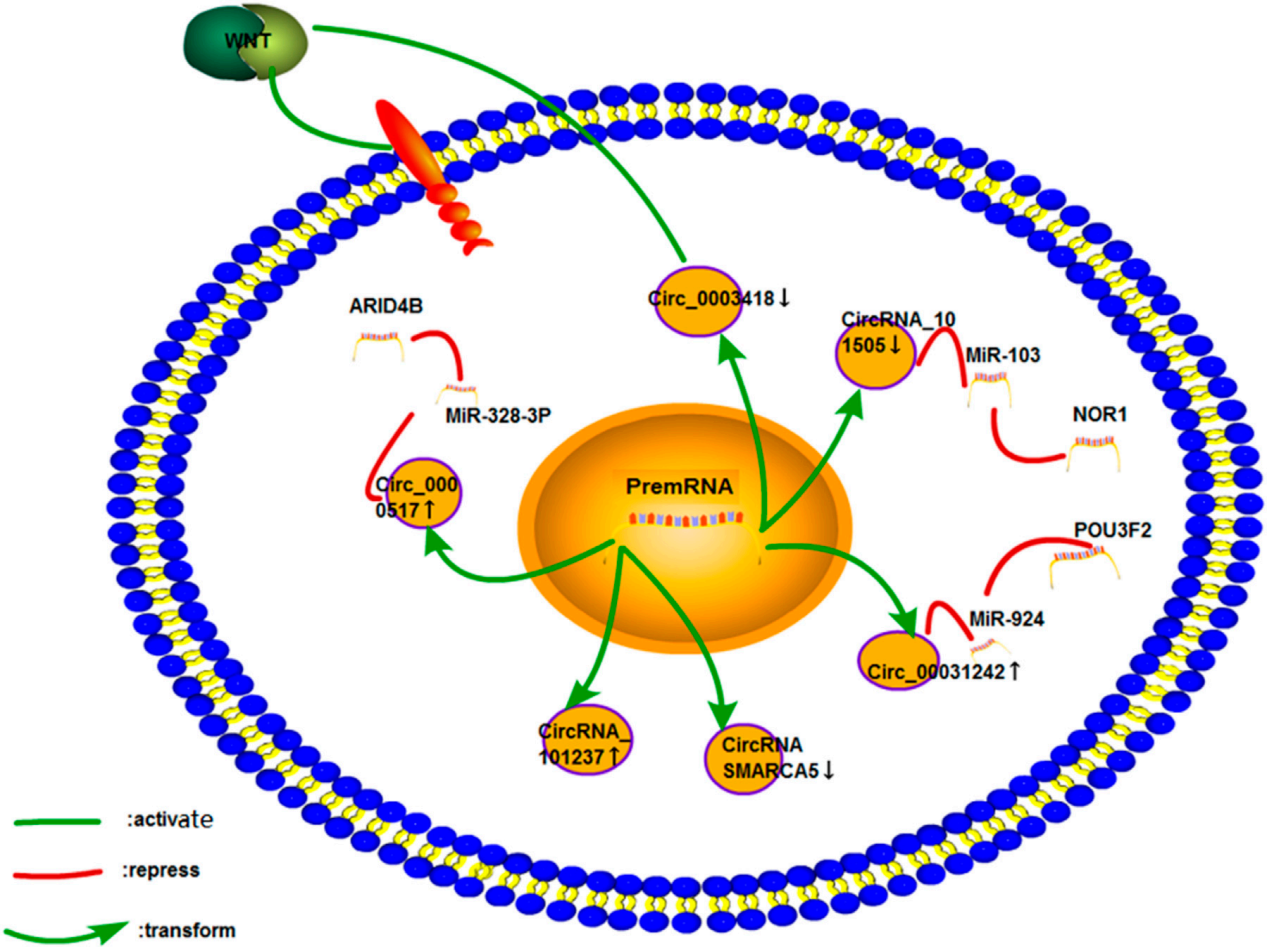
Cisplatin resistance mechanism of circRNA in liver cancer. Only six studies on liver cancer have been performed, and few studies on the mechanism of drug resistance have been conducted. The mechanism of the cisplatin resistance of CircRNA_101237 and CircRNA SMARCA5 has not been studied.

Liver cancer is the second leading cause of cancer death worldwide (Pratama et al., 2019). Cisplatin is the standard drug for transcatheter arterial chemoembolization in the treatment of liver cancer in Japan (Ikeda, 2019). However, cisplatin resistance often occurs during chemotherapy and consequently lowers the survival rate of patients (Chen et al., 2019b). (Figure 12).

Hepatobiliary carcinoma (HCC) is the most common among all liver cancers (Chen et al., 2019b). Yanwei Luo et al. found a new circRNA (circRNA_101505) that was downregulated in the cisplatin resistance of HCC. Bioinformatic prediction and luciferase assay revealed that miR-103 was a direct downstream target of circRNA_101505. CircRNA_101505 could enhance the sensitivity of cisplatin to liver cancer cell lines through miR-103/NOR1 (Luo et al., 2019). Wei Fan et al. used qRT-PCR and Western blot analysis to examine the gene and protein expression levels of circ_0031242, miR-924, and POU3F2. They found that the miR-924/POU3F2 axis could be regulated to promote the cisplatin resistance, proliferation, and invasion of hepatoma cells by circ_0031242. The same results were observed in xenografts (Fan et al., 2021). The upregulation of the circ_0000517 expression in cisplatin-resistant hepatocellular carcinoma cells could promote cell cycle arrest and apoptosis through the miR-328-3P/ARID4B axis. The effect of circ_0000517 on glycolysis in liver cancer cells was analyzed through an ECAR assay. Circ_0000517 could reduce cell glycolysis (Zhao et al., 2021).

Circ_0003418 was found to lower cisplatin resistance in HCC. Silencing circ_0003418 could enhance cisplatin resistance in HCC via the Wnt/catenin signaling pathway (Chen et al., 2019b). Shuping Zhou et al. showed that circRNA_101237 was not only related to the proliferation, invasion, metastasis, and stage of HCC; it also enhanced the resistance of HCC to cisplatin (Zhou et al., 2020). However, the exact drug resistance mechanism of circRNA_101237 remains unclear.

Intrahepatic cholangiocarcinoma is the second-most common type of primary cancer of the liver. Qi Lu et al. conducted a subgroup analysis on 92 patients with intrahepatic cholangiocarcinoma. They found that the expression of circ-SMARCA5 in cancer tissues decreased, and this was negatively correlated with ECOG grading and TNM stage. The overexpression of circ-SMARCA5 could increase the sensitivity of ICC cells to cisplatin and gemcitabine (Lu and Fang, 2020).

### Osteosarcoma

**FIGURE 13:**
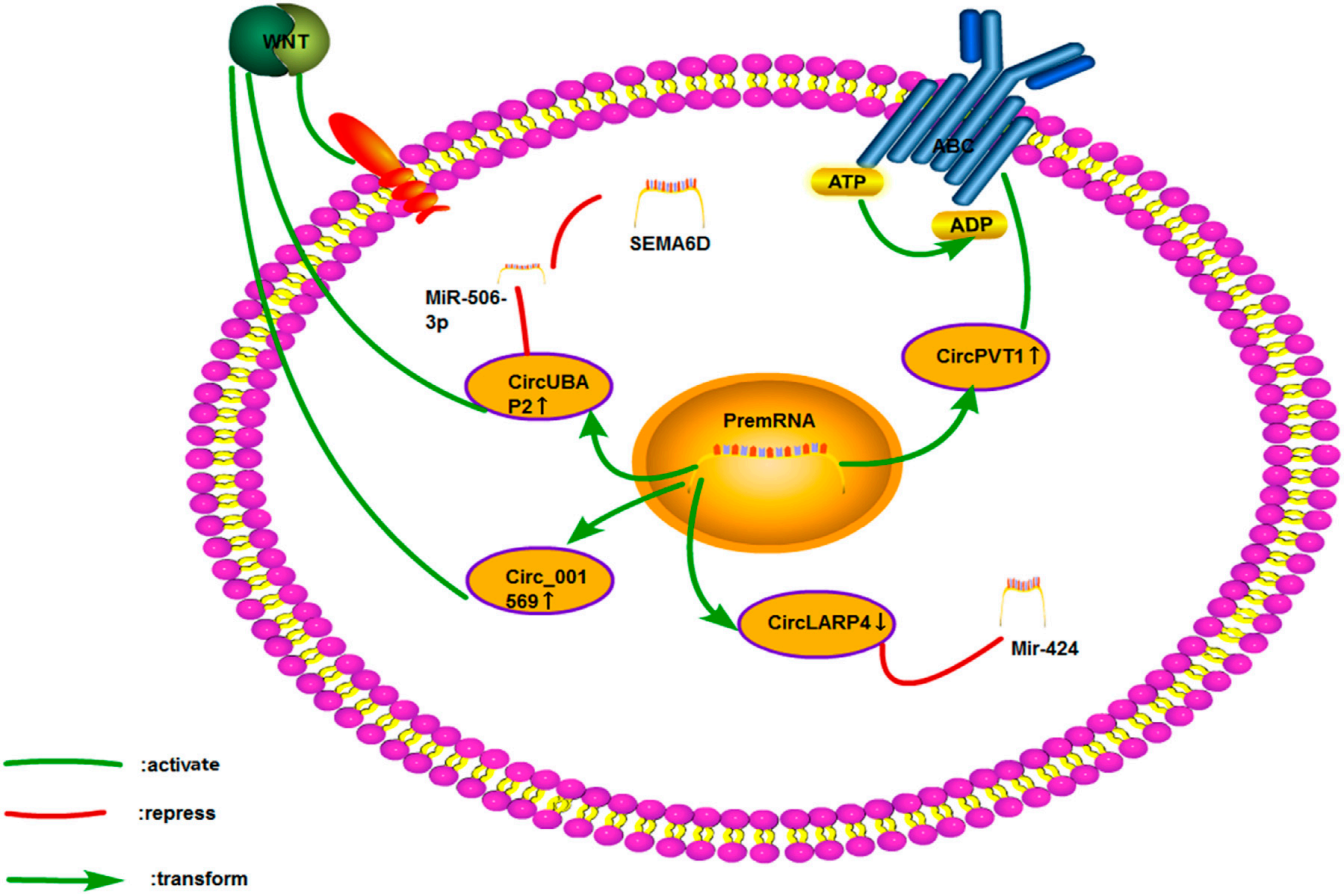
Cisplatin resistance mechanism of circRNA in osteosarcoma. Only four papers have been published on the cisplatin resistance of circRNA in osteosarcoma, which involves ceRNA NET, Wnt signaling pathway, and ABC transporter.

Osteosarcoma is one of the most common malignant bone tumors in young individuals (Zhang et al., 2018b; Gong et al., 2020b; Hu et al., 2020). The treatment regime covers a few weeks of chemotherapy before and after surgery. The main treatment method includes methotrexate, adriamycin, and cisplatin (Tang et al., 2019). (Figure 13).

Yuhang Hu et al. evaluated 72 patients with osteosarcoma. They found that circRNA LARP4 was negatively correlated with the Enneking staging of osteosarcoma. In osteosarcoma cell lines with overexpressed circRNA LARP4, the IC_50_ value related to cisplatin and adriamycin was significantly lowered. Similarly, the circRNA LARP4 could increase its sensitivity to chemotherapy by sponging miR-424 (Hu et al., 2020). Recent studies had shown that the circRNA LARP4 could promote the sensitivity of breast cancer to adriamycin (Zhang et al., 2020c). Through the miR-506-3P/SEMA6D axis, circUBAP2 was reported to activate the Wnt/catenin signaling pathway and promote the proliferation, invasion, and cisplatin resistance of osteosarcoma. The expression of SEMA6D was upregulated in GC and esophageal cancer. However, few studies have investigated its drug resistance mechanism (Gong et al., 2020b).

Circ_001569 was also associated with the Wnt signaling pathway in osteosarcoma. Upregulating the expression of circ_001569 in osteosarcoma was correlated with distant metastasis and TNM staging, and the resistance of osteosarcoma cells to cisplatin was enhanced by activating the Wnt/catenin signaling pathway (Zhang et al., 2018b). As mentioned earlier, circPVT1 could regulate the expression of ABCC1 and promote cisplatin resistance in lung cancer (Zheng and Xu, 2020). Zhu Kun-Peng et al. found that circPVT1 could promote the resistance of osteosarcoma to cisplatin and adriamycin. Silencing circPVT1 was found to lower the expression of the drug resistance-related gene ABCB1 (Kun-Peng et al., 2018).

### Thyroid Cancer

Thyroid cancer is a common malignant tumor (Liu et al., 2018a). It can be divided into five pathological types: papillary thyroid carcinoma (PTC), follicular thyroid cancer, poorly differentiated thyroid cancer, medullary thyroid carcinoma, and undifferentiated thyroid cancer (Liu et al., 2018a; Liu et al., 2018b). The most aggressive type is anaplastic thyroid cancer (ATC) (Zhang et al., 2014). The treatment of thyroid cancer includes surgery, radioactive iodine therapy, and chemotherapy (Carling and Udelsman, 2014). Cisplatin is the foundation of chemotherapy, but its effect is unsatisfactory, especially for ATC (Zhang et al., 2014).

Liu et al. confirmed that circEIF6 (HSA) could enhance the cisplatin resistance of cells in PTC and ATC. CircEIF6 regulates the TGF-β expression by sponging miR-144-3P, promotes the autophagy of thyroid cancer cells, and enhances cisplatin resistance (Liu et al., 2018b). TGF-β is an important gene in EMT mechanism. EMT may participate in the above process. However, the author did not discuss it.

### Cervical Carcinoma/Ovarian Carcinoma

Cervical carcinoma is the second-most common malignant tumor in women, and ovarian carcinoma is the most common cause of death in females (Vaughan et al., 2011; Peng et al., 2016). Cisplatin is still the main chemotherapy regimen in both cancers (Vargas-Hernández et al., 2014; Feng et al., 2017). However, cisplatin resistance has affected the curative effect and survival rate of patients (Waldmann et al., 2013; Peng et al., 2016).

The differential expression of circRNA CDR1as in bladder cancer and NSCLC had been reported. circRNA CDR1as was highly expressed in NSCLC cells, whereas it is weakly expressed in bladder cancer cells (Yuan et al., 2019; Zhao et al., 2020). Zhao et al. recently found that circRNA CDR1as was also poorly expressed in ovarian carcinoma and could enhance the sensitivity of cisplatin through the miR-1270/SCAI axis (Zhao et al., 2019). Therefore, circRNA CDR1as has a huge potential in the research on cisplatin resistance. In terms of exosomes, studies have explored ovarian carcinoma. Luo Yanwei Luo et al. Found that high levels of the exosomal circFOXP1 in cisplatin-resistant ovarian carcinoma could regulate the expression of CEBPG and FMNL3 through miR-22 and miR-150-3P to improve the cisplatin resistance of ovarian carcinoma (Luo and Gui, 2020).

Few studies have been performed on cervical cancer. In one study, Hsa_circ_0023404 could promote the invasion, metastasis, and cisplatin resistance of cervical cancer through the miR-5047/VEGFA axis. In addition, using the autophagy inhibitor 3-MA could recover the inhibitory effect of hsa_circ_0023404 on autophagy-induced apoptosis and improve the sensitivity of chemotherapy (Guo et al., 2019).

### Nasopharyngeal Carcinoma

Nasopharyngeal carcinoma is a common head and neck cancer worldwide (He et al., 2015). Cisplatin is still the main chemotherapeutic drug in the standard chemotherapy regimen for patients with advanced nasopharyngeal carcinoma (Tang et al., 2018). However, numerous patients have a poor response to chemotherapy, resulting in cisplatin resistance (Liu et al., 2018c).

Qiongna Dong et al. found that the overexpression of hsa_circ_002807 was related to the staging, invasion, and metastasis of nasopharyngeal carcinoma, and hsa_circ_002807 could enhance the resistance to cisplatin and paclitaxel (Qiongna et al., 2020). However, its mechanism remains to be elucidated. The effect of circRNAs on cisplatin resistance in nasopharyngeal carcinoma in terms of autophagy should be further investigated.

### Oral Squamous Cell Carcinoma

OSCC is a highly invasive malignant tumor and the most common malignant tumor in the head and neck (Bray et al., 2018; Pai et al., 2019). Surgery, postoperative radiotherapy, and chemotherapy are the main treatment methods. The main drug in chemotherapy is cisplatin (Pai et al., 2019). However, the emergence of chemotherapy resistance decreases the curative effect of chemotherapeutic drugs (Pérez-Sayáns et al., 2010).

Circ_0001971 had recently been found to increase significantly in OSCC. Circ_0001971 could promote the proliferation, invasion, migration, and cisplatin resistance of tumors by sponging miR-194 and miR-204. Western blot analysis showed that the EMT-related proteins cyclin D1 and N-cadherin and the expression of E-cadherin increased significantly after silencing circ_0001971 (Tan et al., 2020). Therefore, EMT plays an important role in it. ABC transporters participate in the cisplatin resistance of OSCC. The expression of circ_0109291 in cisplatin-resistant OSCC cell lines was upregulated, thereby inhibiting apoptosis. Circ_0109291 could promote cisplatin resistance via the miR-188-3P/ABCB1 axis (Gao et al., 2020).

### Laryngocarcinoma

Laryngocarcinoma is one of the most common cancers in the respiratory system, head, and neck (Liu et al., 2016; Steuer et al., 2017). It is more common in men than in women (Steuer et al., 2017). Its main treatment strategy is chemotherapy, especially patients at an advanced stage (Liu et al., 2016). However, drug resistance occurs with the long-term use of chemotherapeutic drugs (Liang et al., 2014). One of the most common chemotherapeutic drugs for laryngocarcinoma is cisplatin. Its initial effect is good, but cisplatin resistance occurs over time (Tian et al., 2017).

Xuehan Yi et al. found that circ_0004507 could promote the proliferation, invasion, migration, and cisplatin resistance of laryngocarcinoma cells by sponging miR-873. Circ_0004507 was revealed to enhance the expression and translation of MDR1 (ABCB1) and ABCC1, so as to regulate cisplatin resistance (Yi et al., 2020). Recently, circPGAM1 had been reported to inhibit apoptosis of laryngocarcinoma cells and enhance cisplatin resistance. MiR-376A was a direct downstream target of circPGAM1, which could inhibit the expression of ATG2A to improve the sensitivity of cisplatin (Feng et al., 2021).

### Carcinoma of the Esophagus

In recent years, the mortality of patients with esophageal cancer has increased significantly. Patients with esophageal cancer are always diagnosed at an advanced stage in addition to the high incidence rate, so chemotherapy becomes important (Huang and Yu, 2018). The basic drug used in chemotherapy is cisplatin (Su et al., 2019c). Cisplatin remains effective at early stages, but the emergence of cisplatin resistance gradually decreases the survival of patients with esophageal cancer (Hou et al., 2017; Zou et al., 2020).

CircRNA_001275 was significantly increased in cisplatin-resistant esophageal cancer tissues and cells. Through the miR-370-3P/WNT7A axis, circRNA_001275 could inhibit apoptosis and promote the proliferation and drug resistance of esophageal cancer cells (Zou et al., 2020). The mechanism of cisplatin resistance in esophageal cancer remains unknown, but it should be further explored in terms of autophagy and signaling pathways.

### Colon Cancer

Colon cancer is the third-most common cancer in men and the second-most common cancer in women, accounting for about 10 and 9.2%, respectively (Pan et al., 2017). The combination of surgery, chemotherapy, and radiation has been the most common treatment for rectal cancer. Chemotherapy is used before, during, and after surgery and has a different role (Karpisheh et al., 2019). Patients with advanced colon cancer are still treated with cisplatin-based combination chemotherapy and radiotherapy to improve the quality of life and prolong the survival time (Scott et al., 2009).

The luciferase experiment showed a targeting relationship between miR-487a-3P and circ_0020095. The *in vitro* and *in vivo* silencing of circ_0020095 could inhibit colon cancer proliferation and invasion and enhance cisplatin sensitivity via the miR-487A-3P/SOX9 axis (Sun et al., 2020b). This finding also provides further insights into targeted treatments for colon cancer and even rectal cancer.

## Conclusion

### Summary and Outlook

Research on circRNA in cisplatin resistance has attracted increasing attention in recent years, and most studies are based on the ceRNA network. The ceRNA network is essential for studying the mechanism of cisplatin resistance. The ceRNA network gives us a good framework in studies. In this large framework, one or several mechanisms involve the combined action in other mechanisms to explore tumor proliferation, invasion, and drug resistance. For example, studies on exosomes should consider whether CSCs participate in this process and detect CSC-related pathways, such as FGFR and MEK, or the mRNA level of related genes (SOX2, OCT4, and Nanog). In addition, the expression of RAS gene, ABCG2 (ABC transporter) and REV3L (DNA repair) may be a future research direction. Autophagy can promote and inhibit tumor proliferation and growth, which are a two-way mechanism. However, recent research has shown that circRNA can promote drug resistance in cisplatin resistance by activating autophagy. In the future, studies should detect the expression of more ATGs, such as P62 and LC3, and explore whether they can inhibit drug resistance. The exosome PD-L1 influences the cisplatin resistance of lung cancer by affecting the immune system. This finding suggests that immunotherapy combined with chemotherapy may improve the postoperative survival rate of patients with cancer. N6-methyladenosine (m6A), a common RNA including ncRNAs (circRNA) methylation modification, has gradually entered the field of vision in the proliferation of tumor cells. However, the role of circRNA m6A in the regulation of chemotherapeutic drugs such as cisplatin still need to be explored further. The cisplatin resistance mechanism of circRNA in different cancers is summarized in this review to provide a basis for future studies on circRNAs. Therefore, this review may provide a new idea for the clinical improvement of cisplatin resistance Table 1.

**TABLE 1:** Collection of papers on circRNA in cisplatin resistance. Expression refers to the circRNA expression in cancers. Targets correspond to targeted genes in circRNA downstream. Mechanism denotes the drug resistance mechanism of circRNA in various tumors. NA: not applicable.

CircRNAs	References	Expression	Targets	Mechanisms	Cancers
Circ_0076305	Dong et al. (2019)	↑	MiR-296-5p/STAT3	CeRNA NET/ABC	Lung cancer
Hsa_circ_0001946	Huang et al. (2019b)	↓	NA	DNA damage repair	Lung cancer
Circ-ABCB10	Wu et al. (2020)	↑	MiR-556-3p/AK4	CeRNA NET	Lung cancer
Circ_0072083	Li et al. (2020b)	↑	MiR-545-3p/CBLL1	CeRNA NET	Lung cancer
Hsa_circ_0085131	Kong, (2020)	↑	MiR-654-5P/ATP7	CeRNA NET/Autophagy	Lung cancer
Circ_0007385	Ye et al. (2020)	↑	MiR-519d-3p	CeRNA NET	Lung cancer
Circ-CPA4	Hong et al. (2020)	↑	Let-7/PD-L1	CeRNANET/EMT/ Exosomes /CSCs	Lung cancer
Hsa_circ_0096157	Huang et al. (2019b)	↑	NA	NA	Lung cancer
CircRNA PRMT5	Pang et al. (2020)	↑	MiR-4458/REV3L	CeRNA/DNA damage repair	Lung cancer
Hsa_circ_0046264	Liu et al. (2020a)	↑	NA	NA	Lung cancer
Circ_0000079	Chen et al. (2020a)	↑	FXR1/PRCKI	EMT	Lung cancer
Circ-SMARCA5	Tong (2020)	↓	NA	NA	Lung cancer
CircCDR1as	Zhao et al. (2020)	↑	MiR-641/HOXA9	CeRNA NET/CSCs	Lung cancer
CircCDR1as	Mao and Xu (2020)	↑	NA	PI3K	Lung cancer
CircPVT1	Zheng and Xu (2020)	↑	MiR-145-5p/ABCC1	CeRNA NET/ABC	Lung cancer
CircPVT1	Lu (2020)	↑	NA	NA	Lung cancer
CircAKT3	Xu et al. (2020a)	↑	MiR-516b-5p/STAT3	CeRNA NET	Lung cancer
Circ-PIP5K1A	Shao et al. (2021)	↑	MiR-101/ABCC1	CeRNA NET/ABC	Lung cancer
Hsa_circ_0081143	Xue et al. (2019a)	↑	MiR-646/CDK6	CeRNA NET	Gastric cancer
CircRNA AKT3	Huang et al. (2019a)	↑	MiR-198/PIK3R1	CeRNANET/DNA damage repair	Gastric cancer
Circ_0110805	Yang et al. (2020a)	↑	MiR-299-3p/ENDOPDI	CeRNA NET	Gastric cancer
CircPVT1	Yao (2020)	↑	MiR-30a-5p/YAP1	CeRNANET/ Exosomes/Autophagy	Gastric cancer
Circ_0026359	Zhang et al. (2020a)	↑	MiR-1200/POLD4	CeRNA NET	Gastric cancer
Circ_0000260	Liu et al. (2020c)	↑	MiR-129-5p/MMP11	CeRNA NET/ Exosomes	Gastric cancer
CircRNA CCDC66	Zhang et al. (2020b)	↑	MiR-618/BCL2	CeRNA NET	Gastric cancer
CircFN1	Huang et al. (2020)	↑	MiR-182-5p	CeRNA NET	Gastric cancer
CircDONSON	Liu et al. (2020b)	↑	MiR-802/BMI1	CeRNA NET	Gastric cancer
CircCUL2	Peng et al. (2020)	↑	MiR-142-3p/ROCK2	CeRNA NET/ Autophagy	Gastric cancer
CircMCTP2	Sun et al. (2020a)	↓	MiR-99a-5p/MTMR3	CeRNANET/Autophagy	Gastric cancer
Hsa_circ_0000285	Chi et al. (2019)	↓	NA	NA	Bladder cancer
CircCDR1as	Yuan et al. (2019)	↓	MiR-1270/APAF1	CeRNA NET	Bladder cancer
CircELP3	Su et al. (2019b)	↑	NA	CSCs	Bladder cancer
CircFNTA	Chen et al. (2020b)	↑	MiR-370-3P/FNTA	CeRNA NET/KARS	Bladder cancer
Hsa_circRNA_102336	Gong et al. (2020a)	↑	MiR-515-5p	CeRNA NET/ABC	Bladder cancer
Circ_0003418	Chen et al. (2019b)	↓	NA	Wnt	Liver cancer
CircRNA_101505	Luo et al. (2019)	↓	MiR-103/NOR1	CeRNA NET	Liver cancer
CircRNA_101237	Zhou et al. (2020)	↑	NA	NA	Liver cancer
CircRNA SMARCA5	Lu and Fang, (2020)	↓	NA	NA	Liver cancer
Circ_0000517	Zhao et al. (2021)	↑	MiR-328-3P/ARID4B	CeRNA NET	Liver cancer
Circ_00031242	Fan et al. (2021)	↑	MiR-924/POU3F2	CeRNA NET	Liver cancer
CircRNA_001275	Zou et al. (2020)	↑	MiR-370-3p/Wnt-7a	CeRNA NET	Esophageal cancer
CircRNA LARP4	Hu et al. (2020)	↓	MiR-424	CeRNA NET	Osteosarcoma
CircUBAP2	Gong et al. (2020b)	↑	MiR-506-3p/SEMA6D	CeRNA NET/Wnt	Osteosarcoma
CircPVT1	Kun-Peng et al. (2018)	↑	NA	ABC	Osteosarcoma
Circ_001569	Zhang et al. (2018b)	↑	NA	Wnt	Osteosarcoma
CircRNA EIF6	Liu et al. (2018b)	↑	MiR-144-3p/TGF-β	CeRNANET/Autophagy	Thyroid cancer
CircFoxp1	Luo and Gui, (2020)	↑	MiR-22/miR-150-3P/CEBPG/FMNL3	CeRNA NET/Exosomes	Ovarian cancer
CircCDR1as	Zhao et al. (2019)	↓	MiR-1270/SCAI	CeRNA NET	Ovarian cancer
Hsa_circ_0023404	Guo et al. (2019)	↑	MiR-5047/VEGFA	CeRNA NET/Autophagy	Cervical cancer
Circ_0004507	Yi et al. (2020)	↑	MiR-573/ABCB1	CeRNA NET/ABC	Laryngeal cancer
CircPGAM1	Feng et al. (2021)	↑	MiR-376a	CeRNA NET	Laryngeal cancer
Circ_0109291	Gao et al. (2020)	↑	MiR-188-3p/ABCB1	ABC	Oral squamous cell carcinoma
Circ_0001971	Tan et al. (2020)	↑	MiR-194/miR-204	CeRNA NET/EMT	Oral squamous cell carcinoma
Hsa_Circ_0028007	Qiongna et al. (2020)	↑	NA	NA	Nasopharyngeal cancer
Hsa_Circ_0020095	Sun et al. (2020b)	↑	MiR-48a-3p/SOX9	CeRNA NET	Colon cancer

## Glossary

ABC: ATP-binding cassette
AR: androgen receptor
ATC: anaplastic thyroid cancer
BCRP: breast cancer resistance protein
CCL2: CC-chemokine ligand 2
CeRNA NET: competing endogenous RNA network
CircRNA: circular RNA
CRC: colorectal cancer
CSCs: cancer stem cells
DDR: DNA damage repair
EGFR: epidermal growth factor receptor
EMT: epithelial–mesenchymal transition
ERCC1: excision repair cross-complementing 1
FGFR: fibroblast growth factor receptor
FDA: Food and Drug Administration
GC: gastric cancer
HCC: hepatocellular carcinoma
Hh: Hedgehog
LncRNA: long noncoding
m6A: N6-methyladenosine
MBL: muscle blind
MDR: multidrug resistance
MDR1: multidrug resistance protein 1
MEK: mitogen-activated protein kinase
MiRNA/MiR: microRNA
MREs: miRNA reaction elements
MRP1: multidrug resistance-related protein 1
NcRNA: noncoding RNA
NER: nucleotide excision repair
OS: overall survival
OSCC: oral squamous cell carcinoma
PD1: programmed cell death protein-1
PD-L1: programmed cell death-ligand 1
PE: phosphatidylethanolamine
PI3K: phosphatidylinositol 3-kinase
PTC: papillary thyroid carcinoma
QRT-PCR: quantitative reverse transcription PCR
SBP: substrate-binding proteins
TGF-β: transforming growth factor-β
TME: tumor microenvironment
